# A Hybrid of the Chemical Master Equation and the Gillespie Algorithm for Efficient Stochastic Simulations of Sub-Networks

**DOI:** 10.1371/journal.pone.0149909

**Published:** 2016-03-01

**Authors:** Jaroslav Albert

**Affiliations:** Applied Physics Research Group, Vrije Universiteit Brussel, Brussels, Belgium; University of Edinburgh, UNITED KINGDOM

## Abstract

Modeling stochastic behavior of chemical reaction networks is an important endeavor in many aspects of chemistry and systems biology. The chemical master equation (CME) and the Gillespie algorithm (GA) are the two most fundamental approaches to such modeling; however, each of them has its own limitations: the GA may require long computing times, while the CME may demand unrealistic memory storage capacity. We propose a method that combines the CME and the GA that allows one to simulate stochastically a part of a reaction network. First, a reaction network is divided into two parts. The first part is simulated via the GA, while the solution of the CME for the second part is fed into the GA in order to update its propensities. The advantage of this method is that it avoids the need to solve the CME or stochastically simulate the entire network, which makes it highly efficient. One of its drawbacks, however, is that most of the information about the second part of the network is lost in the process. Therefore, this method is most useful when only partial information about a reaction network is needed. We tested this method against the GA on two systems of interest in biology - the gene switch and the Griffith model of a genetic oscillator—and have shown it to be highly accurate. Comparing this method to four different stochastic algorithms revealed it to be at least an order of magnitude faster than the fastest among them.

## Introduction

In a network of chemical reactions, the molecular concentrations at any given time cannot be predicted with a certainty; they can only be anticipated with a certain probability. This probability can in principle be determined by solving (analytically or numerically) the chemical master equation (CME). Attempting to do so, however, can more often than not be a frustrating exercise: except for a handful of simple cases, the CME cannot be solved analytically, and for a lot of interesting cases even a numerical solution can be near impossible to attain. One way around this obstacle was an algorithm proposed by Doob [1] and later presented and popularized by Gillespie [2]. The authors showed that the information stored in the CME can be extracted through a series of relatively simple steps coupled with the help of a pseudo-random number generator. Known today by its popular name as the Gillespie algorithm (GA) (also known as kinetic Monte Carlo or stochastic simulation algorithm (SSA)), this procedure guarantees an exact solution to the CME, provided these steps are repeated sufficiently many times so as to build a statistically significant ensemble of data points. The solution to the CME can thus be reconstructed step by step without the need for enormous memory storage capacity that is usually required to solve the CME directly. The one drawback of the GA is that the number of steps required scales with the number of reactions and the magnitude of their rates. Consequently, for large reaction networks the running time may become impractical.

Since it first appeared, researchers have devised faster versions of the GA, some of which are exact [3, 4], in the sense that they give statistically identical answers as the CME, while others rely on approximations [5–8]. In conjunction with the Langevin approximation [9], these algorithms comprise a library of methods to chose from when simulating reaction networks. The Dizzy package [10], for instance, is one such library containing four stochastic simulators of various speed and accuracy.

With the advent of stochastic algorithms, the appeal for solving the CME directly has not diminished however. Having an analytical solution to the CME, even an approximate one, is extremely useful and can provide insight into the stochastic properties of chemically interacting networks. It is not surprising then that many methods for finding approximate solutions to the CME exist and continue to appear [11–16].

There exists a class of methods which involve partitioning a reaction network in a way that facilitates either a solution to the CME [17, 18] or a faster stochastic simulation [19–21] of one part of the reaction network, while yielding only a partial information about the rest of the network. Other partitioning methods rely on large differences among the values of the reaction rates [22, 23]. Such methods take advantage of the fast rates by considering the chemical species affected by these rates to be in a quasi-steady state.

In this paper, we present another method where synergy between the CME and the GA is exploited for the purpose of simulating one part of a reaction network. While previous methods of this kind rely on *a priory* approximations of either the CME or the GA, we begin by deriving the exact equations from which the next reaction time and the next reaction probability can be computed, as well as the exact CME for the non-simulated part of the network. Once these equations take form, they may be solved by virtue of approximations, either as a matter of necessity or in order to speed up the simulation process. We show on two biologically relevant examples how this method can be applied and discuss what its limitations are.

## Materials and Methods

### Chemical Master Equation

The time evolution of the joint probability distribution *P*(**n**, *t*) for a chemically reacting system comprising *N* molecular species and *J* reactions is governed by the chemical master equation (CME):

$$\begin{array}{c} \dot{P}\left(n,t\right)=\sum_{\mu =1}^{J}a_{\mu }\left(n-\upsilon_{\mu }\right)P\left(n-\upsilon_{\mu },t\right)-P\left(n,t\right)\sum_{\mu =1}^{J}a_{\mu }\left(n\right), \end{array}$$ (1)

where **n** is short for the set {*n*_1_, *n*_2_, …, *n*_*N*_}, and *a*_1_(**n**)…*a*_*J*_(**n**), are the reaction propensities, which, for our purposes here, will depend on time only explicitly, i. e. through the variables **n**. The matrix elements *υ*_*iμ*_ specify the change in *n*_*i*_ due to the *μ*th reaction. It will be useful later on to represent Eq (1) in another way [9, 24–26]. Let us define a vector state

$$\begin{array}{c} |\psi \left(t\right)\rangle =\sum_{n_{1},n_{2}\ldots n_{N}}P\left(n,t\right)\prod_{i=1}^{N}|n_{i}\rangle , \end{array}$$ (2)

and operators ${\hat{A}}_{i}$, ${\hat{A}}_{i}^{†}$ and ${\hat{n}}_{i}$ the action of which on the state |*n*_*i*_〉 is

$$\begin{array}{c} {\hat{A}}_{i}|n_{i}\rangle =n_{i}|n_{i}-1\rangle ,\;\;\;\;\;\;\;{\hat{A}}_{i}^{†}|n_{i}\rangle =|n_{i}+1\rangle ,\;\;\;\;\;\;\;{\hat{n}}_{i}|n_{i}\rangle =n_{i}\left|n_{i}\right\rangle , \end{array}$$ (3)

where, by definition, ${\hat{n}}_{i}={\hat{A}}_{i}^{†}{\hat{A}}_{i}$. The index *i* means that the operators act only on the *i*th vector state, leaving the rest of them untouched. The vector state |*n*_*i*_〉, and its transpose 〈*n*_*i*_| are simply the orthogonal unit column and row vectors respectively:

$$\begin{array}{c} |n_{i}\rangle ={\left(\begin{array}{c} 0\\ .\\ .\\ 0\\ 1\\ 0\\ .\\ .\\ . \end{array}\right)}_{i}\;\;\;\;\;\;\;\;\;\;\left\langle n_{i}\right|={\left(\begin{array}{cccccccc} 0, & . & . & 1, & 0, & . & . & . \end{array}\right)}_{i} \end{array}$$ (4)

so that 〈0| = (1, 0, 0, …), 〈1| = (0, 1, 0, …), etc. The operators ${\hat{A}}_{i}$ and ${\hat{A}}_{i}^{†}$ have the form

$$\begin{array}{c} {\hat{A}}_{i}={\left[\begin{array}{cccccc} 0 & 1 & 0 & 0 & . & .\\ 0 & 0 & 2 & 0 & & \\ 0 & 0 & 0 & 3 & & \\ . & & & . & & \\ . & & & & . & \\ . & & & & & . \end{array}\right]}_{i}\;\;\;\;\;\;\;\;\;\;{\hat{A}}_{i}^{†}={\left[\begin{array}{cccccc} 0 & 0 & 0 & . & . & .\\ 1 & 0 & 0 & & & \\ 0 & 1 & 0 & & & \\ 0 & 0 & 1 & . & & \\ . & & & & . & \\ . & & & & & . \end{array}\right]}_{i}. \end{array}$$ (5)

The vector state product in Eq (2) can be thought of as a vector whose elements are the individual vector states, |*n*_*i*_〉:

$$\begin{array}{c} \prod_{i=1}^{N}\left|n_{i}\right\rangle =\left(\begin{array}{c} |n_{1}\rangle \\ |n_{2}\rangle \\ .\\ .\\ .\\ |n_{N}\rangle \end{array}\right). \end{array}$$ (6)

A product of any operators, e. g. ${\hat{A}}_{1}{\hat{A}}_{2}$, could then be represented by an *N* × *N* matrix:

$$\begin{array}{c} {\hat{A}}_{1}{\hat{A}}_{2}={\hat{A}}_{1}{\hat{A}}_{2}1_{3}...1_{N}=\left[\begin{array}{cccccc} {\hat{A}}_{1} & 0 & 0 & . & . & .\\ 0 & {\hat{A}}_{2} & 0 & 0 & & \\ 0 & 0 & 1_{3} & 0 & & \\ . & & & . & & \\ . & & & & . & \\ . & & & & & 1_{N} \end{array}\right], \end{array}$$ (7)

where $1_{i}$ is the identity operator: $1_{i}|n_{i}\rangle =|n_{i}\rangle$.

With this notation, the master equation can be written in the form

$$\begin{array}{c} \frac{d}{dt}\left|\psi \left(t\right)\right\rangle =\hat{H}\left|\psi \left(t\right)\right\rangle , \end{array}$$ (8)

where

$$\begin{array}{c} \hat{H}=\sum_{\mu =1}^{J}\left[\prod_{j=1}^{N}{\left({\left({\hat{n}}_{j}+1\right)}^{-1}{\hat{A}}_{j}\right)}^{\theta \left(-\upsilon_{j\mu }\right)\left|\upsilon_{j\mu }\right|}{\left({\hat{A}}_{j}^{†}\right)}^{\theta \left(\upsilon_{j\mu }\right)\left|\upsilon_{j\mu }\right|}-\prod_{j=1}^{N}1_{j}\right]a_{\mu }\left(\hat{n}\right) \end{array}$$ (9)

is an operator acting on the vector state $\prod_{i=1}^{N}|n_{i}\rangle$ and *θ*(.) is a step function centered around zero.

Let us check that Eqs (8) and (1) are indeed identical. To see how the operator $\hat{H}$ in Eq (9) acts on the state |*ψ*〉, let us first look at how the operators within it act on the individual vector states |*n*_*j*_〉. We have

$$\begin{array}{ccc} {\left({\left({\hat{n}}_{j}+1\right)}^{-1}{\hat{A}}_{j}\right)}^{a}{\left({\hat{A}}_{j}^{†}\right)}^{b}\left|n_{j}\right\rangle & = & {\left({\left({\hat{n}}_{j}+1\right)}^{-1}{\hat{A}}_{j}\right)}^{a-1}\left[\left({\left({\hat{n}}_{j}+1\right)}^{-1}{\hat{A}}_{j}\right)\right]\left|n_{j}+b\right\rangle \\ & = & {\left({\left({\hat{n}}_{j}+1\right)}^{-1}{\hat{A}}_{j}\right)}^{a-1}\left[\left(n_{j}+b\right){\left({\hat{n}}_{j}+1\right)}^{-1}\right]\left|n_{j}+b-1\right\rangle \\ & = & {\left({\left({\hat{n}}_{j}+1\right)}^{-1}{\hat{A}}_{j}\right)}^{a-1}\left|n_{j}+b-1\right\rangle \\ & = & {\left({\left({\hat{n}}_{j}+1\right)}^{-1}{\hat{A}}_{j}\right)}^{a-2}\left|n_{j}+b-2\right\rangle \\ & = & {\left({\left({\hat{n}}_{j}+1\right)}^{-1}{\hat{A}}_{j}\right)}^{a-3}\left|n_{j}+b-3\right\rangle \\ & \cdot & \\ & \cdot & \\ & \cdot & \\ & = & |n_{j}+b-a\rangle \end{array}$$ (10)

and hence,

$$\begin{array}{c} {\left({\left({\hat{n}}_{j}+1\right)}^{-1}{\hat{A}}_{j}\right)}^{\theta \left(-\upsilon_{j\mu }\right)\left|\upsilon_{j\mu }\right|}{\left({\hat{A}}_{j}^{†}\right)}^{\theta \left(\upsilon_{j\mu }\right)\left|\upsilon_{j\mu }\right|}|n_{j}\rangle =|n_{j}+\theta \left(\upsilon_{j\mu }\right)|\upsilon_{j\mu }|-\theta \left(-\upsilon_{j\mu }\right)|\upsilon_{j\mu }\left|\right\rangle . \end{array}$$ (11)

Since the second term is merely the identity operator, it leaves the state unchanged. The operator $a_{\mu }\left(\hat{n}\right)$ acting on the product state ∏_*j*_|*n*_*j*_〉 also leaves it unchanged and itself becomes a number, *a*_*μ*_(**n**). Putting the above relations together, we can write

$$\begin{array}{ccc} \hat{H}\left|\psi \right\rangle & = & \sum_{n_{1},n_{2}...n_{N}}P\left(n,t\right)\hat{H}\left[\prod_{j=1}^{N}\left|n_{j}\right\rangle \right]\\ & = & \sum_{n_{1},n_{2}...n_{N}}\sum_{\mu =1}^{J}P\left(n,t\right)a_{\mu }\left(n\right)\left[\prod_{j=1}^{N}|n_{j}+\theta \left(\upsilon_{j\mu }\right)|\upsilon_{j\mu }|-\theta \left(-\upsilon_{j\mu }\right)|\upsilon_{j\mu }\left|\right\rangle -\prod_{j=1}^{N}\left|n_{j}\right\rangle \right]\\ & = & \sum_{n_{1},n_{2}...n_{N}}\sum_{\mu =1}^{J}P\left(n-\upsilon_{\mu },t\right)a_{\mu }\left(n-\upsilon_{\mu }\right)\left[\prod_{j=1}^{N}\left|n_{j}\right\rangle \right], \end{array}$$ (12)

where in the last line we used the fact that *υ*_*jμ*_ = *θ*(*υ*_*jμ*_)|*υ*_*jμ*_|−*θ*(−*υ*_*jμ*_)|*υ*_*jμ*_|. The left hand side of Eq (8) states that

$$\begin{array}{c} \frac{d}{dt}\left|\psi \left(t\right)\right\rangle =\sum_{n_{1},n_{2}...n_{N}}\frac{d}{dt}P\left(n,t\right)\left[\prod_{j=1}^{N}\left|n_{j}\right\rangle \right]. \end{array}$$ (13)

The only way expressions Eqs (12) and (13) can be equal is if the coefficients of the product vector state ∏_*j* = 1_|*n*_*j*_〉 on both sides are equal. This leads to Eq (1).

The formal solution to Eq (8) is

$$\begin{array}{c} \left|\psi \left(t\right)\right\rangle =e^{\hat{H}t}\left|\psi \left(0\right)\right\rangle , \end{array}$$ (14)

where |*ψ*(0)〉 is the initial vector state specified entirely by *P*(**n**, 0). The operator $e^{\hat{H}t}$ is called the evolution operator. Multiplying both sides of Eq (14) by 〈**n**| and invoking the orthogonality relation $\langle n^{\prime }|n\rangle =\delta_{n_{1}^{\prime }n_{1}}\delta_{n_{2}^{\prime }n_{2}}...\delta_{n_{N}^{\prime }n_{N}}$ we obtain the probability distribution

$$\begin{array}{c} \left\langle n|\psi \left(t\right)\right\rangle =\langle n|e^{\hat{H}t}|\psi \left(0\right)\rangle =P\left(n,t\right), \end{array}$$ (15)

where for brevity $|n\rangle =\prod_{i=1}^{N}|n_{i}\rangle$. With this formalism it is easy to write down quantities such as the transition probabilities. For example,

$$\begin{array}{c} W\left(n^{\prime },t^{\prime }|n,t\right)=\langle n^{\prime }|e^{\hat{H}\left(t^{\prime }-t\right)}\left|n\right\rangle , \end{array}$$ (16)

means the probability to find the system in the state **n**′ at time *t*′ if at time *t* it was in the state **n**.

Lastly, let us also write down the identity operator in the form

$$\begin{array}{c} 1=\sum_{n}\left|n\rangle \langle n\right| \end{array}$$ (17)

as it will be useful in later sections.

### Gillespie Algorithm

The idea behind the GA is to simulate a chain of Markov processes by sampling the probability distribution of the time elapsed since the last reaction, *τ*, and the probability that a specific reaction, *μ*, will occur at *τ*, such that any reaction occurring at *τ* has probability 1. The steps are as follows:

1. At some initial time *t* (e. g. *t* = 0) select your initial state **n** and compute the propensities *a_μ_*(**n**).
2. Select two random numbers *r*_1_ and *r*_2_.
3. Compute *τ* using the formula
  $$\begin{array}{c} \tau =\left(1/a_{0}\right)\ln\left(1/r_{1}\right), \end{array}$$ (18)
  where $a_{0}=\sum_{\mu }a_{\mu }\left(n\right)$.
4. Find the smallest integer *j* that satisfies
  $$\begin{array}{c} \sum_{j^{\prime }=1}^{j}a_{j^{\prime }}\left(n\right)>r_{2}a_{0}\left(n\right), \end{array}$$ (19)
  and set *j* = *μ*.
5. Update the system according to $n\left(t+\tau \right)=n\left(t\right)+\upsilon_{\mu }$ and set *t* = *t* + *τ*.
6. Return to step 1.

Repeating these steps until *t* reaches some final time leads to a particular path, or realization, for **n**. In order to obtain the same information within this time interval as is contained in the CME, one must compute this realization infinitely many times. It is in this sense that the GA and the CME are exactly equivalent. Of course, in practice one only needs to compute a finite number of realizations to extract meaningful information about the system. Depending on how many realizations are considered “sufficient” and how long it takes to compute each realization, the GA may be a fast route to solving the CME, or it may be a very slow one. In the next section we will show how one can combine the CME and the GA in order to simulate stochastically a part of a system.

### CME-GA hybrid

Consider a reaction network of *J* reactions with propensities {*a*_1_, …, *a*_*J*_}, comprising two sets of molecular species: $m=\{m_{1},...,m_{N_{1}}\}$ and $n=\{n_{1},...,n_{N_{2}}\}$ (*N*_1_ + *N*_2_ = *N*). The propensities are some functions of **m** and **n**, but not time. Let us arrange the reactions into two groups, *G*_1_ = {*a*_1_, …, *a*_*K*−1_} and *G*_2_ = {*a*_*K*_, …, *a*_*J*_}, such that the reactions in group *G*_1_ can affect both **m** and **n**, while the reactions in group *G*_2_ can only affect **n**. During a time interval, *τ*, in which no reaction occurs in *G*_1_, the CME for *G*_2_ can be written as follows:

$$\begin{array}{ccc} {\dot{P}}_{m}\left(n,t\right)= & & \sum_{\mu =K}^{J}a_{\mu }\left(m,n-\upsilon_{\mu }\right)P_{m}\left(n-\upsilon_{\mu },t\right)\\ & & -P_{m}\left(n,t\right)\sum_{\mu =K}^{J}a_{\mu }\left(m,n\right). \end{array}$$ (20)

The subscripts **m** in *P*_m_(**n**, *t*) serve as a reminder that the solution of *P*_m_(**n**, *t*) will depend on their value. Note that by definition **n** may change during *τ* only via the reaction channels in *G*_2_, but not in *G*_1_. Let us now see how one can sample *τ* and the next reaction in *G*_1_ from their respective probability distributions in a manner similar to the one described in the previous section. We begin by deriving the probability distribution for *τ*.

Let us divide time into *L* discrete infinitesimal intervals, Δ*t*, such that Δ*tL* = *τ*. Using the notation introduced earlier, the probability that no reaction in *G*_1_ occurs in the time *τ* can be expressed as

$$\begin{array}{ccc} Q(\tau ) & = & \sum_{n_{0},n_{1},...}P_{m}\left(n_{0},0\right)\prod_{k=0}^{L-1}e^{-\Sigma_{k}\Delta t}\times \\ & & \langle n_{L}|e^{\hat{H}\Delta t}|n_{L-1}\rangle \langle n_{L-1}|e^{\hat{H}\Delta t}|n_{L-2}\rangle ...\langle n_{2}|e^{\hat{H}\Delta t}|n_{1}\rangle \langle n_{1}|e^{\hat{H}\Delta t}\left|n_{0}\right\rangle , \end{array}$$ (21)

where $\Sigma_{k}=\sum_{i=K}^{J}a_{i}\left(m,n_{k}\right)$ and *k* refers to the *k*th time interval. The exponential terms, exp[−Σ_*k*_ Δ*t*], are the probabilities that, starting at time *t*_*k*_ = *k*Δ*t*, no reaction occurs in the time Δ*t*. Since the Σs are numbers, not operators, we can move them wherever we want within the total product. Especially useful is to move each exp[−Σ_*k*_ Δ*t*] just left of the state |**n**〉 for each *k*:

$$\begin{array}{ccc} Q(\tau ) & = & \sum_{n_{1},n_{2},...}\langle n_{L}|e^{\hat{H}\Delta t}e^{-\Sigma_{L-1}\Delta t}|n_{L-1}\rangle \langle n_{L-1}|e^{\hat{H}\Delta t}e^{-\Sigma_{L-2}\Delta t}\left|n_{L-2}\right\rangle ...\\ & & \;\;\;\;\;\;\;\;\;\;...\langle n_{2}|e^{\hat{H}\Delta t}e^{-\Sigma_{1}\Delta t}|n_{1}\rangle \langle n_{1}|e^{\hat{H}\Delta t}e^{-\Sigma_{0}\Delta t}\left|\phi \right\rangle , \end{array}$$ (22)

where $|\phi \rangle =\sum_{n_{0}}P_{m}\left(n_{0},0\right)|n_{0}\rangle$ is the initial state. Now, thanks to the fact that

$$\begin{array}{c} \Sigma_{k}|n_{k}\rangle =\sum_{i=1}^{K}a_{i}\left(m,n_{k}\right)|n_{k}\rangle ={\hat{\Sigma }}_{k}|n_{k}\rangle =\sum_{i=1}^{K}a_{i}\left(m,{\hat{n}}_{k}\right)\left|n_{k}\right\rangle , \end{array}$$ (23)

and the relation $\lim_{\epsilon \to 0}\text{exp}\left[\hat{B}\epsilon \right]\text{exp}\left[\hat{C}\epsilon \right]=\text{exp}\left[\left(\hat{B}+\hat{C}\right)\epsilon \right]$ for arbitrary operators $\hat{B}$ and $\hat{C}$, we may write

$$\begin{array}{ccc} Q(\tau ) & = & \sum_{n_{L}}\left\langle n_{L}\right|e^{{\hat{H}}^{\prime }\Delta t}\left(\sum_{n_{L-1}}|n_{L-1}\rangle \langle n_{L-1}|\right)e^{{\hat{H}}^{\prime }\Delta t}\left(\sum_{n_{L-2}}|n_{L-2}\rangle \langle n_{L-2}|\right)...\\ & & \;\;\;\;\;\;\;...e^{{\hat{H}}^{\prime }\Delta t}\left(\sum_{n_{1}}|n_{1}\rangle \langle n_{1}|\right)e^{{\hat{H}}^{\prime }\Delta t}\left|\phi \right\rangle =\sum_{n_{L}}\langle n_{L}|e^{{\hat{H}}^{\prime }t_{L}}\left|\phi \right\rangle , \end{array}$$ (24)

where ${\hat{H}}^{\prime }=\hat{H}+\hat{\Sigma }$. Recalling Eq (17), we can set all the terms in the parentheses to unity. The expression for *Q*(*τ*) can now be written as

$$\begin{array}{c} Q\left(\tau \right)=\sum_{n_{L}}Q_{m}\left(n_{L},t_{L}\right), \end{array}$$ (25)

where, setting *t*_*L*_ to *τ*,

$$\begin{array}{c} Q_{m}\left(n,\tau \right)=\left\langle n\right|e^{{\hat{H}}^{\prime }\tau }\left|\phi \right\rangle . \end{array}$$ (26)

This expression is identical in structure to Eq (15) and as such can be expressed in a differential form:

$$\begin{array}{ccc} {\dot{Q}}_{m}\left(n,t\right)= & & \sum_{\mu =1}^{K-1}a_{\mu }\left(n-\upsilon_{\mu }\right)Q_{m}\left(n-\upsilon_{\mu },t\right)\\ & & -Q_{m}\left(n,t\right)\sum_{\mu =1}^{J}a_{\mu }\left(n\right), \end{array}$$ (27)

with the initial conditions that *Q*_m_(**n**, 0) = *P*_m_(**n**, 0). This equation resembles Eq (20) except that now we have all propensities, from *G*_1_ and *G*_2_, appearing in the second term.

Next we need to compute the probability *p*_*μ*_ that the *μ*th reaction in *G*_1_ occurs at time *τ*. This is given by the probability that the *μ*th reaction occurs given a specific set **n**, multiplied by the probability of having **n**, and then summing over all **n**. In symbols:

$$\begin{array}{c} p_{\mu }=\sum_{n_{1},...,n_{N}}P_{m}\left(n,\tau \right)\frac{a_{\mu }\left(m_{0},n\right)}{a(m_{0},n)},\;\;\;\;\;\;\;\;\;\;a\left(m_{0},n\right)=\sum_{\nu =1}^{K-1}a_{\nu }\left(m_{0},n\right). \end{array}$$ (28)

We now have everything we need to simulate the evolution of **m** via the CME-GA. Here are the steps:

1. Select your initial set **m** and initial probability *P*_m_(**n**, 0) and hence *Q*_m_(**n**, 0) (since *P*_m_(**n**, 0) = *Q*_n_(**n**, 0)).
2. Solve Eqs (20) and (27) and compute *Q*(*τ*) and *p*_*μ*_ for *μ* = 1, …, *K* − 1 according to Eqs (25) and (28).
3. Compute *τ* and select the next reaction *μ* according to:
  i Generate a random real number *ξ*_1_ in the range [0, 1] and solve *ξ*_1_ = *Q*(*τ*) for *τ*
  ii Generate another random real number *ξ*_2_ in the range [0, 1] and select the smallest integer *k* that satisfies the condition $\sum_{j=1}^{k}p_{j}>\xi_{2}$. Set *μ* = *k*.
4. Update **m** and let *P*_m_(**n**, *τ*) be the new initial condition for Eqs (20) and (27) if and only if the selected reaction does not effectuate a change in *n*_*i*_. If the selected reaction changes an *n*_*i*_ by ±*w*_*i*_ (*w*_*i*_ = 1, 2…), the new initial probability *P*_m_(**n**, *τ*) must be modified according to:
  $$\begin{array}{c} P_{m}\left(n,\tau \right)\to P_{m}{\left(n,\tau \right)|}_{n_{i}=n_{i}-w_{i}}\;\;\;\;\;\;\;\;\text{if}\;n_{i}\to n_{i}+w_{i} \end{array}$$
  $$\begin{array}{c} P_{m}\left(n,\tau \right)\to {\hat{L}}_{i}P_{m}\left(n,\tau \right)\;\;\;\;\;\;\;\;\text{if}\;n_{i}\to n_{i}-w_{i}, \end{array}$$
  where ${\hat{L}}_{i}$ is an operator that transforms *P*_m_(**n**, *τ*) like so:
  $$\begin{array}{ccc} {\hat{L}}_{i}P_{m}\left(n,\tau \right) & = & [P_{m}{\left(n,\tau \right)|}_{n_{i}=0}+P_{m}{\left(n,\tau \right)|}_{n_{i}=w_{i}}{]\delta_{n_{i}0}+\sum_{j=1}^{w_{i}-1}P_{m}\left(n,\tau \right)|}_{n_{i}=j}\delta_{n_{i}j}\\ & & +P_{m}{\left(n,\tau \right)|}_{n_{i}=n_{i}+w_{i}}\prod_{j=0}^{w_{i}-1}\left(1-\delta_{n_{i}w_{i}}\right). \end{array}$$
  If more than one species of *n* is affected by a reaction in *G*_1_, the above transformation must be applied to all of them, e. g. ${\hat{L}}_{1}{\hat{L}}_{2}...P_{m}\left(n,\tau \right)$.

This procedure allows one to simulate stochastically a part of a reaction network, i. e. **m**, at the expense of losing information about the rest of the network, i. e. **n**. However, the tractability of this algorithm will depend on the system of interest and on the way in which it is partitioned. If, for instance, a particular choice of partition leads to Eqs (20) and/or (27) being too complicated to solve efficiently, the speed of this algorithm may end up being inferior to other stochastic algorithms. Another obstacle to efficiency is having to solve *ξ*_1_ = *Q*(*τ*) for *τ*, which, depending on the particulars of *P*(*τ*), might be a difficult task. One way to solve for *τ* is to use a minimization algorithm that optimizes the measure (*ξ*_1_ − *Q*(*τ*))^2^ with respect to *τ*. This, however, will likely require a number of steps, calling into question the efficiency of this algorithm. Same can be said of Eq (28), in which the summation(s) may or may not have a closed form. These potential difficulties require not only that the system be partitioned wisely, but also that some of the steps above be simplified/approximated. In the next section we will test the CME-GA on two biological systems and see how it may be applied effectively and accurately.

## Results

### The genetic switch

**Fig 1:**
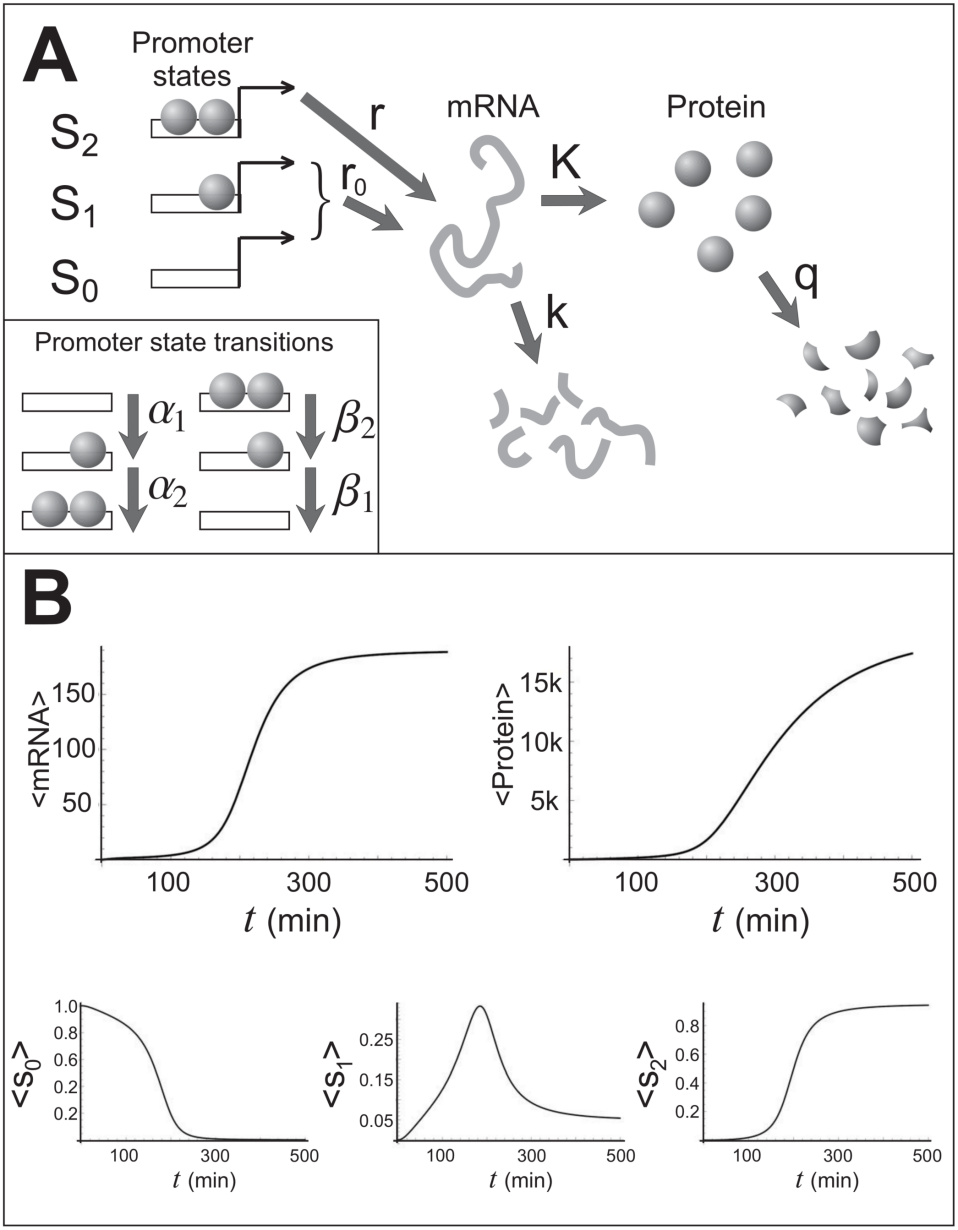
The genetic switch: reactions and dynamics. **A**) A schematic of the genetic switch. B) Dynamics of average mRNA, *m*, protein, *n*, and the three states of promoter, *S*_0_, *S*_1_ and *S*_2_. The chosen reaction frequencies in inverse minutes were: *α*_1_ = *α*_2_ = 0.001, *β*_1_ = *β*_2_ = 1, *r*_0_ = 0.1, *r* = 10, *K* = 1, *k* = 0.05, *q* = 0.01.

Let us consider a single-gene motif with positive autoregulation and a promoter cooperativity of 2. This system can exhibit very large noise [27] due to its positive feedback, and is therefore of interest in systems biology. The simplest yet realistic version of this gene motif is the one described by the following reactions (see Fig 1A):

$$\begin{array}{ccc} S_{0}+n & \overset{\alpha_{1}S_{0}n}{\to } & S_{1}\;\;\;\;\;\;\;\;\;\;\;\;\;\;\;\;\;\;\;\;\;\;\;\;\;\;\;\;\;\;\;\;\;\;\;\;m\overset{Km}{\to }m+n\\ S_{1} & \overset{\beta_{1}S_{1}}{\to } & S_{0}+n\;\;\;\;\;\;\;\;\;\;\;\;\;\;\;\;\;\;\;\;\;\;\;\;\;\;\;n\overset{qn}{\to }\phi \\ S_{1}+n & \overset{\alpha_{2}S_{1}n}{\to } & S_{2}\\ S_{2} & \overset{\beta_{2}S_{2}}{\to } & S_{1}+n\\ S_{0} & \overset{r_{0}S_{0}}{\to } & S_{0}+m\\ S_{1} & \overset{r_{0}S_{1}}{\to } & S_{1}+m\\ S_{2} & \overset{rS_{2}}{\to } & S_{2}+m\\ m & \overset{km}{\to } & \phi \end{array}$$ (29)

where *m* stands for the copy number of mRNA molecules, *n* for the copy number of proteins, and *S*_0_, *S*_1_ and *S*_2_ label the promoter states: unoccupied, occupied by one protein, and occupied by two proteins, respectively. The reaction propensities appear above each arrow. The parameters *α*_*i*_, *β*_*i*_, *r*, *r*_0_, *K*, *k*, *q* are the reaction frequencies per molecule. One can get a sense for the dynamics of this system by looking at the evolution of its averaged variables, ${\bar{S}}_{0}$, ${\bar{S}}_{1}$, ${\bar{S}}_{2}$, $\bar{m}$ and $\bar{n}$, given by the set of ordinary differential equations (ODE)

$$\begin{array}{ccc} & {\dot{\bar{S}}}_{1}=\alpha_{1}\bar{n}\left(1-{\bar{S}}_{1}-{\bar{S}}_{2}\right)+\beta_{2}{\bar{S}}_{2}-\left(\alpha_{2}{\bar{S}}_{1}\bar{n}+\beta_{1}\right){\bar{S}}_{1}, & \\ & {\dot{\bar{S}}}_{2}=\alpha_{2}{\bar{S}}_{1}\bar{n}-\beta_{2}{\bar{S}}_{2}, & \\ & \dot{\bar{m}}=r{\bar{S}}_{2}+r_{0}-k\bar{m}, & \\ & \dot{\bar{n}}=K\bar{m}-q\bar{n}, & \end{array}$$ (30)

where ${\bar{S}}_{0}=1-{\bar{S}}_{1}-{\bar{S}}_{2}$. Fig 1B shows the dynamics of ${\bar{S}}_{0}$, ${\bar{S}}_{1}$, ${\bar{S}}_{2}$, $\bar{m}$ and $\bar{n}$.

Let us now employ the CME-GA to study the stochastic properties of a part of this system. Notice that the reactions were organized into two columns. The reactions in the left column effectuate a change in either *m* or in both *S*_*i*_ and *n* together, but not exclusively in *n*; the reactions that change *n* only appear in the right column. Hence, referring to the notation in the previous section, the left column represents the set **m** = {*S*_0_, *S*_1_, *S*_2_, *m*}, while the right column represents the set **n** = {*n*}. Hence, the two equations, Eqs (20) and (27), reduce to:

$$\begin{array}{c} {\dot{P}}_{m}\left(n,t\right)=KmP_{m}\left(n-1,t\right)+q\left(n+1\right)P_{m}\left(n+1,t\right)-P_{m}\left(n,t\right)\left(Km+qn\right), \end{array}$$ (31)

and

$$\begin{array}{ccc} {\dot{Q}}_{m}\left(n,t\right) & = & KmQ_{m}\left(n-1,t\right)+q\left(n+1\right)Q_{m}\left(n+1,t\right)\\ & & -Q_{m}\left(n,t\right)\left[\left(\alpha_{1}S_{0}+\alpha_{2}S_{1}\right)n+\beta_{1}S_{1}+\beta_{2}S_{2}+r0+rS_{2}+\left(K+k\right)m+qn\right]. \end{array}$$ (32)

It is easy to check that, provided the initial probability $P_{m}\left(n,0\right)$ is a Poisson distribution, the solution to both, Eqs (31) and (32), is also a Poisson distribution. Thus

$$\begin{array}{c} P\left(n,t\right)=e^{-\lambda (t)}\frac{\lambda {\left(t\right)}^{n}}{n!}, \end{array}$$ (33)

and

$$\begin{array}{c} Q\left(n,t\right)=e^{-g(t)}\frac{h{\left(t\right)}^{n}}{n!}, \end{array}$$ (34)

where for notational simplicity the indexes **m** were omitted. Inserting *P*(*n*, *t*) and *Q*(*n*, *t*) into Eqs (31) and (32) respectively yields

$$\begin{array}{c} \dot{\lambda }=Km-q\lambda , \end{array}$$ (35)

$$\begin{array}{c} \dot{h}=Km-\tilde{q}h, \end{array}$$ (36)

$$\begin{array}{c} \dot{g}=\tilde{K}-qh, \end{array}$$ (37)

where $\tilde{q}=\alpha_{1}S_{0}+\alpha_{2}S_{1}+q$ and $\tilde{K}=\beta_{1}S_{1}+\beta_{2}S_{2}+r_{0}+rS_{2}+\left(K+k\right)m$. With the initial conditions *Q*(*n*, 0) = *P*(*n*, 0), and hence *g*(0) = *h*(0) = *λ*(0), we obtain

$$\begin{array}{c} \lambda \left(t\right)=\left[\lambda \left(0\right)-\frac{Km}{q}\right]e^{-qt}+\frac{Km}{q}, \end{array}$$ (38)

$$\begin{array}{c} h\left(t\right)=\left[\lambda \left(0\right)-\frac{Km}{\tilde{q}}\right]e^{-\tilde{q}t}+\frac{Km}{\tilde{q}}, \end{array}$$ (39)

$$\begin{array}{c} g\left(t\right)=\left[\lambda \left(0\right)-\frac{Km}{\tilde{q}}\right]\left(\frac{q}{\tilde{q}}\right)\left(e^{-\tilde{q}t}-1\right)+\left[\tilde{K}-\frac{Kmq}{\tilde{q}}\right]t+\lambda \left(0\right). \end{array}$$ (40)

The probability distribution for *τ* acquires a closed form:

$$\begin{array}{c} Q\left(\tau \right)=\sum_{n=0}^{\infty }Q\left(n,\tau \right)=e^{-g(\tau )+\lambda (\tau )}. \end{array}$$ (41)

Referring to Eq (28), we can readily compute the probabilities for each reaction in the left column to occur. In the order in which they appear in Eq (29), they read:

$$\begin{array}{ccc} p_{1} & = & S_{0}\left[1-\left\langle \frac{a_{1}}{n+a_{1}}\right\rangle \right]\\ p_{2} & = & S_{1}\frac{\beta_{1}}{\alpha_{2}}\left\langle \frac{1}{n+a_{1}}\right\rangle \\ p_{3} & = & S_{1}\left[1-\left\langle \frac{a_{2}}{n+a_{2}}\right\rangle \right]\\ p_{4} & = & S_{2}\frac{\beta_{2}}{\beta_{2}+r+km}\\ p_{5} & = & S_{0}\frac{r_{0}}{\alpha_{1}}\left\langle \frac{1}{n+a_{1}}\right\rangle +S_{1}\frac{r_{0}}{\alpha_{2}}\left\langle \frac{1}{n+a_{2}}\right\rangle +S_{2}\frac{r}{\beta_{2}+r+km}\\ p_{6} & = & S_{0}\frac{km}{\alpha_{1}}\left\langle \frac{1}{n+a_{1}}\right\rangle +S_{1}\frac{km}{\alpha_{2}}\left\langle \frac{1}{n+a_{2}}\right\rangle +S_{2}\frac{km}{\beta_{2}+r+km}, \end{array}$$ (42)

where

$$\begin{array}{c} a_{1}=\frac{r_{0}+km}{\alpha_{1}},\;\;\;\;\;\;\;\;\;\;\;a_{2}=\frac{\beta_{1}+r_{0}+km}{\alpha_{2}}, \end{array}$$ (43)

and 〈…〉 stands for $\sum_{n=0}^{\infty }P\left(n,\tau \right)\left(...\right)$. To work out the exact expressions for all the *p*s we can employ the formula

$$\begin{array}{c} \sum_{n=0}^{\infty }\frac{b^{n}}{(a+n)n!}=B\left(a,b\right),\;\;\;\;\;\;\;\;\;B\left(a,b\right)={\left(-b\right)}^{-a}\left[\Gamma \left(a\right)-\Gamma \left(a,-b\right)\right], \end{array}$$ (44)

with Γ(.) being the Gamma function. Thus, we have

$$\begin{array}{c} \left\langle \frac{1}{n+a_{i}}\right\rangle =e^{-\lambda (\tau )}B\left(a_{i},\lambda \left(\tau \right)\right). \end{array}$$ (45)

We now have all ingredients to run the CME-GA. However, before we do, let us consider the computational expenses involved in performing all the steps. In particular, solving Eq (41) for *τ* may slow down the algorithm considerably, as it requires an optimization algorithm of some kind. One way to avoid this is to solve Eq (41) approximately by assuming that the solution, i. e. *τ*, is small and expand ln *Q*(*τ*) up to *τ*^2^. The equation for *τ* then becomes

$$\begin{array}{c} \ln\xi_{1}=\ln Q\left(\tau \right)\approx -b_{1}\tau +b_{2}\tau^{2}/2. \end{array}$$ (46)

where

$$\begin{array}{c} b_{1}=\tilde{K}-Km+\left(\tilde{q}-q\right)\lambda \left(0\right),\;\;\;\;\;\;\;\;b_{2}=\left[Km-\tilde{q}\lambda \left(0\right)\right]\left(\tilde{q}-q\right). \end{array}$$ (47)

Writing *τ* = *τ*_0_ + *τ*_1_, where *τ*_0_ satisfies ln1/*ξ* = *b*_1_ *τ*_0_ and *τ*_1_ is a correction, we obtain the ratio

$$\begin{array}{c} \frac{\tau_{1}}{\tau_{0}}=\frac{1}{2}\frac{\left(b_{2}/b_{1}\right)\ln\left(1/\xi_{1}\right)}{b_{1}-\left(b_{2}/b_{1}\right)\ln\left(1/\xi_{1}\right)}. \end{array}$$ (48)

Thus, as long as *τ*_1_/*τ*_0_ < *ϵ*, where *ϵ* is some small number, e. g. 0.001, we may write

$$\begin{array}{c} \tau =\frac{1}{b_{1}}\ln\frac{1}{\xi_{1}}. \end{array}$$ (49)

This equation is identical in structure to Eq (18), especially when we see that *b*_1_ = (*α*_1_ *S*_0_ + *α*_2_ *S*_1_)*λ*(0) + *β*_1_ *S*_1_ + *β*_2_ *S*_2_ + *r*_0_ + *rS*_2_ + *km*, which is just the sum of all reactions in the left column but with *n* replaced by its average, *λ*(0). The condition *τ*_1_/*τ*_0_ < *ϵ* must be incorporated into the CME-GA and checked for each cycle; if it fails, Eq (41) must be solved by some other means, e. g. an optimization algorithm.

Similarly for the reaction probabilities, we may simplify them by expanding 1/(*a*_*i*_ + *n*) around *n* − 〈*n*〉 and then averaging each term:

$$\begin{array}{c} \left\langle \frac{1}{a_{i}+n}\right\rangle \approx \frac{1}{a_{i}+\lambda \left(0\right)}+\frac{\lambda (0)}{{\left(a_{i}+\lambda \left(0\right)\right)}^{3}}-\frac{\lambda (0)}{{\left(a_{i}+\lambda \left(0\right)\right)}^{4}}\cdot \cdot \cdot , \end{array}$$ (50)

where we used the fact that for a Poisson distribution 〈*n*〉 = 〈(*n* − 〈*n*〉)^2^〉 = 〈(*n* − 〈*n*〉)^3^〉, which in the present case is equivalent to *λ*(0). Here again we need to keep in mind that this approximation may become inaccurate (depending on the number of terms), as for instance when *λ*(0), *a*_1_ < 1.

**Fig 2:**
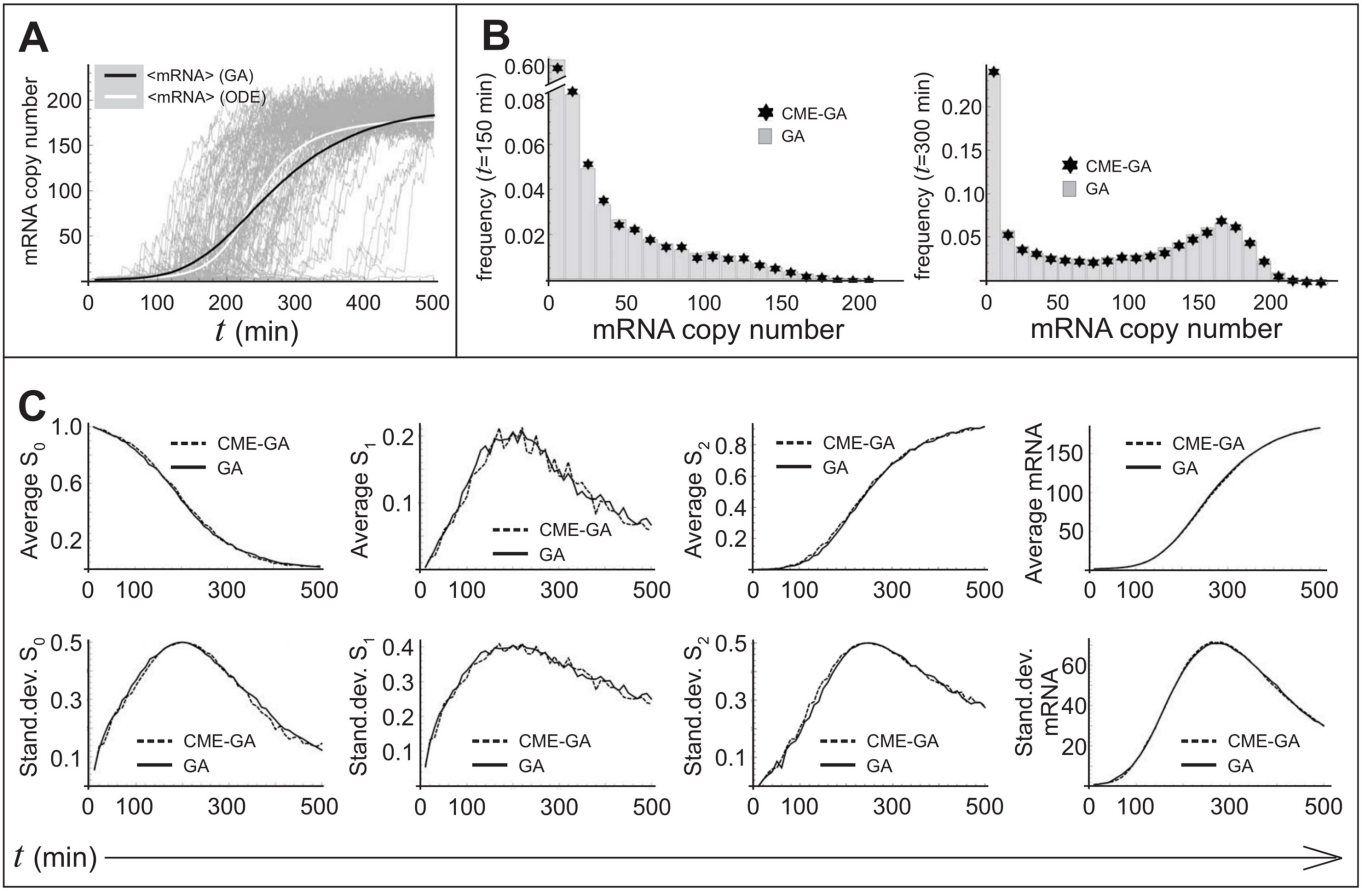
Comparison of the CME-GA with the GA—Genetic switch. **A**) A superposition of 100 realizations generated by the GA. The black solid curve represents the average of 500 realizations, while the white curve is the solution of Eq (30) for $\bar{m}$. B) Probability distributions for *m* at *t* = 150min and *t* = 300min, showing the match between the CME-GA (asterisk) and the GA (bar) constructed from an ensemble size of 10000. C) Comparison between the CME-GA and the GA of the averages and standard deviations of *m*, *S*_0_, *S*_1_ and *S*_2_. The ensemble size was 1000.

Finally, before running the CME-GA, we need to address its forth step. Remember that expressions Eqs (33) and (34) are the solutions to Eqs (31) and (32) only if their initial distributions are Poissonian. However, when reactions 1–4 in the left column occur, we must add to or subtract from the system one copy of *n*, and then modify the new initial probability *P*(*n*, *τ*) according to step 4 of the CME-GA. This however will render Eqs (33) and (34) incorrect. There may be ways to overcome this problem; however, in the present case, with *α*_1_, *α*_2_ < <1, we are justified in ignoring it. Running the CME-SSA with the parameters of Fig 1 leads to the results of Fig 2.

### The Griffith model of a genetic oscillator

**Fig 3:**
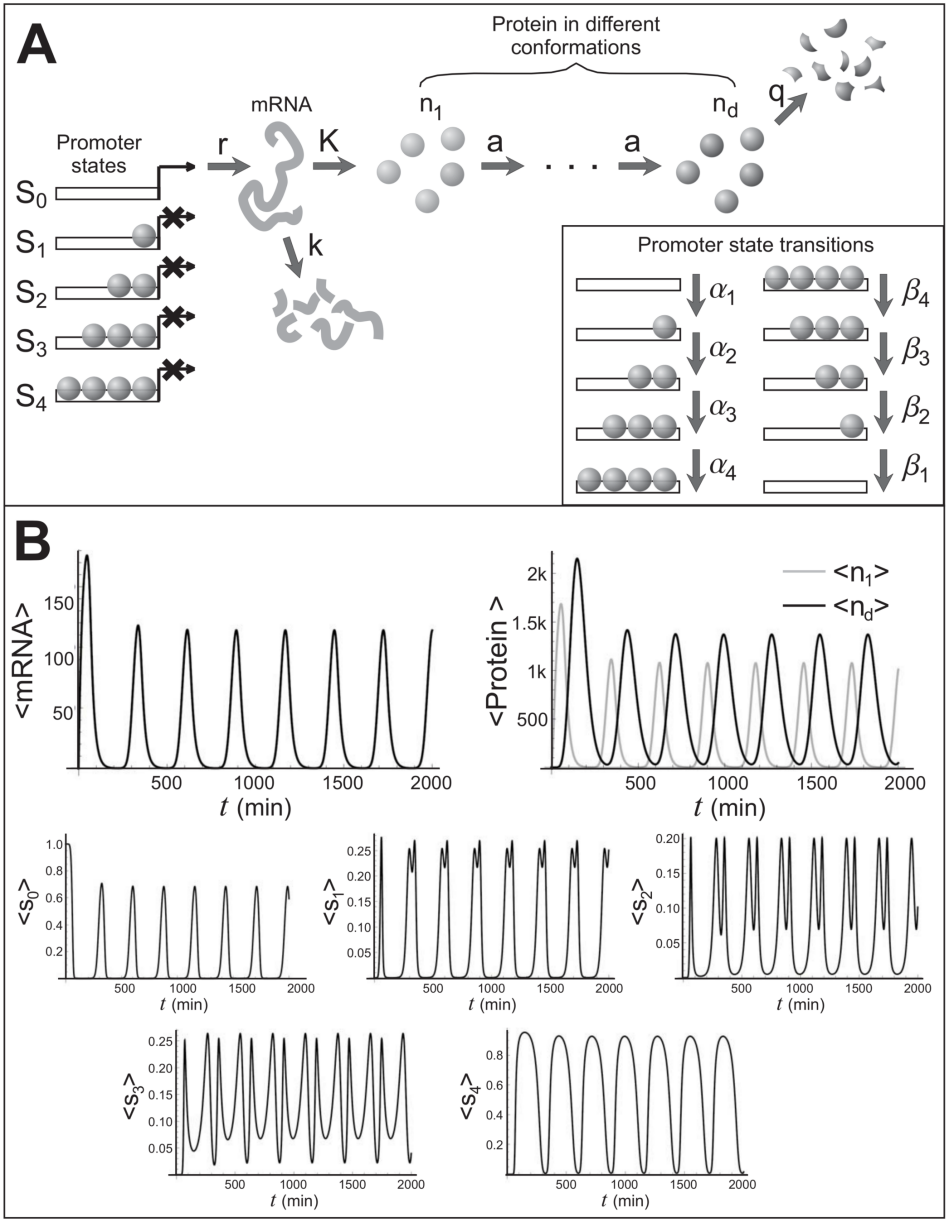
The Griffith model: reactions and dynamics. **A**) A schematic of the Griffith model. B) Dynamics of average mRNA, *m*, protein, *n*, and the five states of promoter, *S*_0_, *S*_1_, *S*_2_, *S*_3_ and *S*_4_. The chosen reaction frequencies in inverse minutes were: *α*_1_ = *α*_2_ = *α*_3_ = *α*_4_ = 0.01, *β*_1_ = *β*_2_ = *β*_3_ = *β*_4_ = 1, *r* = 10, *K* = 1, *k* = 0.05, *q* = 0.05, *a* = 0.1. The number of protein conformations, *d* was set to 10.

Consider now a larger network consisting of a promoter with three states, *S*_0_, *S*_1_, and *S*_2_, an mRNA, and a protein that can be in several conformations, e. g. when undergoing a multi-step phosphorylation [28], such that in its final conformation the protein can bind to its promoter and repress it (see Fig 3A). The reactions for this system are as follows:

$$\begin{array}{ccc} S_{0}+n_{d} & \overset{\alpha_{1}S_{0}n_{d}}{\to } & S_{1}\;\;\;\;\;\;\;\;\;\;\;\;\;\;\;\;\;\;\;\;\;\;\;\;\;\;\;\;\;\;\;\;\;\;\;\;\;m\overset{Km}{\to }m+n_{1}\\ S_{1} & \overset{\beta_{1}S_{1}}{\to } & S_{0}+n_{d}\;\;\;\;\;\;\;\;\;\;\;\;\;\;\;\;\;\;\;\;\;\;n_{1}\overset{an_{1}}{\to }n_{2}\\ S_{1}+n_{d} & \overset{\alpha_{2}S_{1}n_{d}}{\to } & S_{2}\;\;\;\;\;\;\;\;\;\;\;\;\;\;\;\;\;\;\;\;\;\;\;\;\;\;\;\;\;\;\;\;\;\;\;\;\;\;\;\;\;\;\;\;\;\;\;\;\;\;\;\;\;\cdot \\ S_{2} & \overset{\beta_{2}S_{2}}{\to } & S_{1}+n_{d}\;\;\;\;\;\;\;\;\;\;\;\;\;\;\;\;\;\;\;\;\;\;\;\;\;\;\;\;\;\;\;\;\;\;\;\;\;\;\;\cdot \\ S_{2}+n_{d} & \overset{\alpha_{3}S_{2}n_{d}}{\to } & S_{3}\;\;\;\;\;\;\;\;\;\;\;\;\;\;\;\;\;\;\;\;\;\;\;\;\;\;\;\;\;\;\;\;\;\;\;\;\;\;\;\;\;\;\;\;\;\;\;\;\;\;\;\;\;\cdot \\ S_{3} & \overset{\beta_{3}S_{3}}{\to } & S_{2}+n_{d}\;\;\;\;\;\;\;\;\;\;\;\;\;\;\;\;\;\;\;\;\;\;\;\;\;\;\;\;\;\;\;\;\;\;\;\;\;\;\;\cdot \\ S_{3}+n_{d} & \overset{\alpha_{4}S_{3}n_{d}}{\to } & S_{4}\;\;\;\;\;\;\;\;\;\;\;\;\;\;\;\;\;\;\;\;\;\;\;\;\;\;\;\;\;\;\;\;\;\;\;\;\;\;\;\;\;\;\;\;\;\;\;\;\;\;\;\;\;\cdot \\ S_{4} & \overset{\beta_{4}S_{4}}{\to } & S_{4}+n_{d}\;\;\;\;\;\;\;\;\;\;\;\;\;\;\;\;\;\;\;\;\;\;\;\;\;\;\;\;\;\;\;\;\;\;\;\;\;\;\;\cdot \\ S_{0} & \overset{rS_{0}}{\to } & S_{0}+m\;\;\;\;\;\;\;\;\;\;\;\;\;\;\;\;\;n_{d-1}\overset{an_{d-1}}{\to }n_{d}\\ m & \overset{km}{\to } & \phi \;\;\;\;\;\;\;\;\;\;\;\;\;\;\;\;\;\;\;\;\;\;\;\;\;\;\;\;\;\;\;\;\;\;\;\;\;\;n_{d}\overset{qn_{d}}{\to }\phi \end{array}$$ (51)

Here again *m* refers to the mRNA copy number, *n*_*i*_ to the protein copy number, where the indexes *i* = 1, …, *d* label different protein conformations. The differential equations for ${\bar{S}}_{0}$, ${\bar{S}}_{1}$, ${\bar{S}}_{2}$, ${\bar{S}}_{3}$, ${\bar{S}}_{4}$, $\bar{m}$ and ${\bar{n}}_{i}$ read:

$$\begin{array}{ccc} & {\dot{\bar{S}}}_{1}=\alpha_{1}{\bar{n}}_{d}{\bar{S}}_{0}+\beta_{2}{\bar{S}}_{2}-\left(\alpha_{2}{\bar{n}}_{d}+\beta_{1}\right){\bar{S}}_{1}, & \\ & {\dot{\bar{S}}}_{2}=\alpha_{2}{\bar{n}}_{d}{\bar{S}}_{1}+\beta_{3}{\bar{S}}_{3}-\left(\alpha_{3}{\bar{n}}_{d}+\beta_{2}\right){\bar{S}}_{2}, & \\ & {\dot{\bar{S}}}_{3}=\alpha_{3}{\bar{n}}_{d}{\bar{S}}_{2}+\beta_{4}{\bar{S}}_{4}-\left(\alpha_{4}{\bar{n}}_{d}+\beta_{3}\right){\bar{S}}_{3}, & \\ & {\dot{\bar{S}}}_{4}=\alpha_{4}{\bar{n}}_{d}{\bar{S}}_{3}-\beta_{4}{\bar{S}}_{4}, & \\ & \dot{\bar{m}}=r{\bar{S}}_{0}-k\bar{m}, & \\ & {\dot{\bar{n}}}_{1}=K\bar{m}-a{\bar{n}}_{1}, & \\ & {\dot{\bar{n}}}_{2}=a\left({\bar{n}}_{1}-{\bar{n}}_{2}\right), & \\ & \cdot & \\ & \cdot & \\ & \cdot & \\ & {\dot{\bar{n}}}_{d}=a{\bar{n}}_{d-1}-q{\bar{n}}_{d}, & \\ & {\bar{S}}_{0}=1-{\bar{S}}_{1}-{\bar{S}}_{2}-{\bar{S}}_{3}-{\bar{S}}_{4} \end{array}$$ (52)

For some values of its reaction rates, this system can exhibit sustained oscillations, as shown in Fig 3.

The solutions to Eqs (20) and (27) for the right column are products of Poisson distributions,

$$\begin{array}{c} P\left(n,t\right)=\prod_{i=1}^{d}\frac{\lambda_{i}{\left(t\right)}^{n_{i}}}{n_{i}!}e^{-\lambda_{i}\left(t\right)} \end{array}$$ (53)

$$\begin{array}{c} Q\left(n,t\right)=e^{-g(t)}\prod_{i=1}^{d}\frac{h_{i}{\left(t\right)}^{n_{i}}}{n_{i}!}, \end{array}$$ (54)

provided again that their initial distributions are also Poisson. Inserting these into Eqs (20) and (27) leads to differential equations for the *λ*s:

$$\begin{array}{ccc} & {\dot{\lambda }}_{1}=Km-a\lambda_{1}, & \\ & {\dot{\lambda }}_{2}=a\left(\lambda_{1}-\lambda_{2}\right), & \\ & \cdot & \\ & \cdot & \\ & \cdot & \\ & {\dot{\lambda }}_{d}=a\lambda_{d-1}-q\lambda_{d}, \end{array}$$ (55)

and another almost identical set for the *h*s but with *q* replaced with $\tilde{q}=\alpha_{1}S_{0}+\alpha_{2}S_{1}+\alpha_{3}S_{2}+\alpha_{4}S_{3}$, and

$$\begin{array}{c} \dot{g}=\tilde{K}-qh_{d}, \end{array}$$ (56)

with $\tilde{K}=\beta_{1}S_{1}+\beta_{2}S_{2}+\beta_{3}S_{3}+\beta_{4}S_{4}+rS_{0}+\left(K+k\right)m$. The solutions for the *λ*s, *h*s and *g* are

$$\begin{array}{ccc} \lambda_{i}\left(t\right) & = & \frac{mK}{a}-e^{-at}\sum_{j=0}^{i-1}\frac{1}{j!}\left(\frac{mK}{a}-\lambda_{i-j}\left(0\right)\right){\left(at\right)}^{j},\;\;\;\;\;i<d,\\ \lambda_{d}\left(t\right) & = & \sum_{j=0}^{d-2}\frac{e^{-qt}}{j!}\left(\frac{Km}{a}-\lambda_{d-j-1}\left(0\right)\right){\left(\frac{a}{a-q}\right)}^{j+1}\left[\Gamma \left(j+1\right)-\Gamma \left(j+1,\left(a-q\right)t\right)\right]\\ & & \;\;\;\;\;\;\;\;\;\;\;\;\;\;\;\;\;\;\;\;\;\;\;\;\;\;\;\;\;\;\;\;\;\;\;\;\;\;\;\;\;\;\;\;\;\;\;\;\;\;\;\;\;\;\;\;\;\;\;\;\;\;\;\;\;\;\;\;\;\;\;\;\;\;\;\;\;\;\;\;\;\;\;\;\;\;\;\;\;\;\;\;\;\;\;\;\;\;\;\;\;\;\;\;\;\;\;\;\;\;+\frac{Km}{q}+\left(\lambda_{d}\left(0\right)-\frac{Km}{q}\right)e^{-qt},\\ h_{i}\left(t\right) & = & \lambda_{i}\left(t\right){|}_{q\to \tilde{q}},\;\;\;\;\;\;\;\forall i,\\ g(t) & = & Kmt-q\int_{0}^{t}h_{d}\left(t^{\prime }\right)dt^{\prime }. \end{array}$$ (57)

Following the steps detailed in the previous section, we arrive at the same approximation for *τ*:

$$\begin{array}{c} \tau =\frac{1}{b_{1}}\ln\frac{1}{\xi_{1}},\;\;\;\;\;\;\;\;b_{1}=\left(\alpha_{1}S_{0}+\alpha_{2}S_{1}\right)\lambda_{d}\left(0\right)+\beta_{1}S_{1}+\beta_{2}S_{2}+r_{0}+rS_{2}+km, \end{array}$$ (58)

with the same error parameter as in Eq (48), *τ*_1_/*τ*_0_, but now with $b_{2}=\left(\tilde{q}-q\right)\left(a\lambda_{d-1}\left(0\right)-q\lambda_{d}\left(0\right)\right)$.

The propensities *p*_1_ to *p*_10_ are given by

$$\begin{array}{ccc} p_{1} & = & S_{0}\left[1-\left\langle \frac{a_{1}}{n_{d}+a_{1}}\right\rangle \right]\\ p_{2} & = & S_{1}\frac{\beta_{1}}{\alpha_{2}}\left\langle \frac{1}{n_{d}+a_{1}}\right\rangle \\ p_{3} & = & S_{1}\left[1-\left\langle \frac{a_{2}}{n_{d}+a_{2}}\right\rangle \right]\\ p_{4} & = & S_{2}\frac{\beta_{2}}{\alpha_{3}}\left\langle \frac{1}{n_{d}+a_{2}}\right\rangle \\ p_{5} & = & S_{2}\left[1-\left\langle \frac{a_{3}}{n_{d}+a_{3}}\right\rangle \right]\\ p_{6} & = & S_{3}\frac{\beta_{3}}{\alpha_{4}}\left\langle \frac{1}{n_{d}+a_{3}}\right\rangle \\ p_{7} & = & S_{3}\left[1-\left\langle \frac{a_{4}}{n_{d}+a_{4}}\right\rangle \right]\\ p_{8} & = & S_{4}\frac{\beta_{4}}{\beta_{4}+km}\\ p_{9} & = & S_{0}\frac{r_{0}}{\alpha_{1}}\left\langle \frac{1}{n+a_{1}}\right\rangle \\ p_{10} & = & km\left[\frac{S_{4}}{km+\beta_{4}}+\sum_{i=1}^{4}\frac{S_{i-1}}{\alpha_{i}}\left\langle \frac{1}{n_{d}+a_{i}}\right\rangle \right], \end{array}$$ (59)

with

$$\begin{array}{c} a_{1}=\frac{km}{\alpha_{1}},\;\;\;\;\;a_{2}=\frac{km+\beta_{1}}{\alpha_{2}},\;\;\;\;\;a_{3}=\frac{km+\beta_{2}}{\alpha_{3}},\;\;\;\;\;a_{4}=\frac{km+\beta_{4}}{\alpha_{4}},\;\;\;\;\; \end{array}$$ (60)

**Fig 4:**
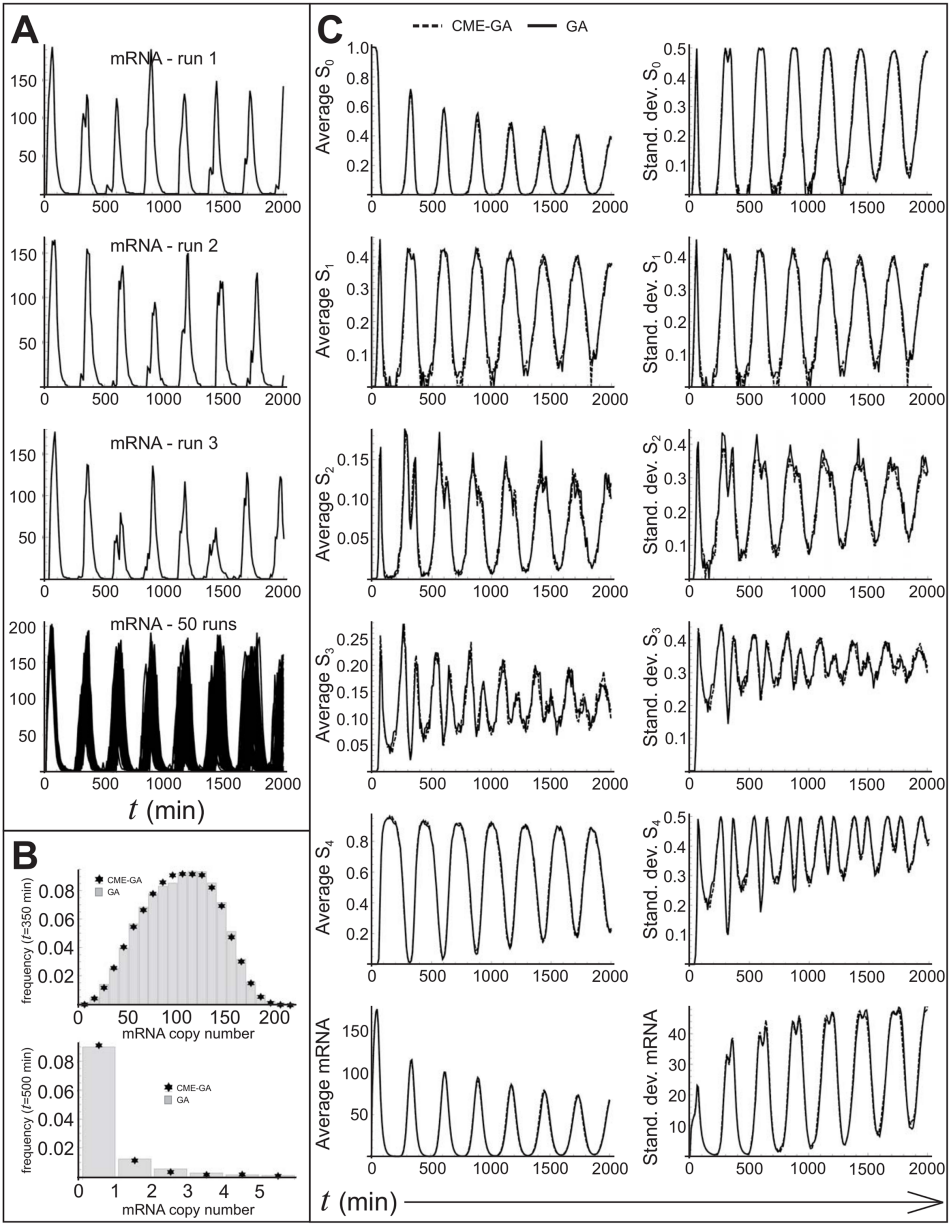
Comparison of the CME-GA with the GA—the Griffith model. **A**) Graphs 1–3 show individual realizations generated by the GA. The forth graph shows a superposition of 50 realizations. B) Probability distributions for *m* at *t* = 350min and *t* = 500min, showing the match between the CME-GA (asterisk) and the GA (bar) constructed from an ensemble size of 10000. C) Comparison between the CME-GA and the GA of the averages and standard deviations of *m*, *S*_0_, *S*_1_, *S*_2_, *S*_3_ and *S*_4_. The ensemble size was 1000.

Finally, making the approximation that reactions *p*_1_-*p*_8_ do not alter *P*_**m**_(**n**, 0) significantly, and expanding the terms 〈…〉 in *n*_*d*_ − *λ*_*d*_(*τ*), we can run the CME-GA. The results are shown in Fig 4.

## Discussion

The method presented herein provides a means of stochastically simulating a reaction sub-network. Because most of the information about the rest of the network is lost, its usefulness is limited to cases where partial information about a network is sufficient. The two examples discussed above illustrate the accuracy of this method. In terms of efficiency, Table 1. shows the computation times of the CME-GA and five other stochastic simulation algorithms that were used to simulate the two models. It is clear that the CME-GA is significantly more efficient than any of the other algorithms.

**Table 1:** State of the art vs. CME-GA.

Algorithm	Genetic switch	Griffith model
Gillespie direct	37	300
Gibson-Bruck	60	297
Tau-leaping simple	60	304
Tau-leaping complex	44	126
CME-GA	2	14

Comparison of computing times (rounded to nearest second) of the four standard algorithms with the CME-GA hybrid. For both, the genetic switch and the Griffith model, the ensemble size was 1000. The stopping time was: 500 for the former and 2000 for the latter.

Important to notice is the relation between the speed of CME-GA and the abundance of those molecular species that appear in the CME Eq (20). Since the speed of the GA scales with the number of species and their abundance, and the CME does not (at least when it can be solved exactly), different partitions will lead to different speeds. If, for instance, we had chosen to partition either of the example systems such that the mRNA appeared in the CME Eq (20) instead of the protein, and it was the protein that was simulated via the GA, the computational time would have been drastically increased. Therefore, a network to be partitioned must be done so wisely. This of course may be in conflict with the user’s desire to simulate a particular set of species. Consequently, if the CME-GA is to remain superior in speed to others, it must be limited to such partitions where the species with large molecular numbers are placed in the non-simulated sub-network.

Although Eqs (20) and (27), which are necessary for the CME-GA to run, were derived exactly, without any assumptions, in practice they may not always be tractable and will require approximations. However, bisecting a system into two groups as proscribed above necessarily renders the CME less complex (Eq (1) vs. Eq (20)) and hence more manageable. It should be noted that as the simulated reaction network grows larger, the time between reactions becomes shorter. This means that Eqs (20) and (27), given a large sub-network (i. e. *G*_1_), will only need to be solved for very short times. Another relief may come from moment closure methods [29–31]: since the propensities in Eq (28) can be expressed as a sum of statistical moments (see Eq (50)), one needs only to solve the set of equations for a few moments, instead of the full CME for the sub-system *G*_2_; and same goes for *Q*_**m**_(**n**, *t*). This last approach might in fact be the most promising way of extending our algorithm to more complex systems.

Lastly, it bears mentioning that although the information about the sub-system *G*_2_ is lost, it may not always be completely lost for all types of systems. In both examples discussed above, when the original CME, Eq (1), is multiplied by the variables in *G*_2_ and summed over all variables, one ends up with

$$\begin{array}{c} \dot{\bar{n}}=K\bar{m}-q\bar{n}, \end{array}$$ (61)

for the genetic switch, and

$$\begin{array}{ccc} {\dot{\bar{n}}}_{1} & = & K\bar{m}-a{\bar{n}}_{1},\\ {\dot{\bar{n}}}_{2} & = & a({\bar{n}}_{1}-{\bar{n}}_{2}),\\ & . & \\ & . & \\ & . & \\ {\dot{\bar{n}}}_{d} & = & a{\bar{n}}_{d-1}-q{\bar{n}}_{d} \end{array}$$ (62)

for the Griffith model. And because $\bar{m}$ is computed via the CME-GA, all the above equations have a closed form.
